# Association between screen time and hyperactive behaviors in children under 3 years in China

**DOI:** 10.3389/fpsyt.2022.977879

**Published:** 2022-11-09

**Authors:** Jian-Bo Wu, Xiao-Na Yin, Shuang-Yan Qiu, Guo-Ming Wen, Wei-Kang Yang, Jing-Yu Zhang, Ya-Fen Zhao, Xin Wang, Xiao-Bing Hong, DaLi Lu, Jin Jing

**Affiliations:** ^1^Shenzhen Longhua Maternity and Child Healthcare Hospital, Shenzhen, China; ^2^Key Laboratory of Brain, Cognition and Education Science, Ministry of Education, Institute for Brain Research and Rehabilitation, Guangdong Key Laboratory of Mental Health and Cognitive Science, South China Normal University, Guangzhou, China; ^3^School of Management, Jinan University, Guangzhou, China; ^4^Department of Maternal and Child Health, School of Public Health, Sun Yat-sen University, Guangzhou, China

**Keywords:** hyperactive behaviors, screen time, early life, boundary, developmental sensitivity

## Abstract

**Background:**

Screen time during early life has increased dramatically among Chinese children. Excessive screen time has raised growing concerns about the neuropsychological development of children. The effects of screen exposure on early life and the boundary between screen time and hyperactive behaviors are well worth investigating. We examined associations between screen time and hyperactive behaviors in children under the age of 3 years using data from the Longhua Children Cohort Study (LCCS).

**Methods:**

A cross-sectional study was conducted among 42,841 3-year-old children from Longhua District, Shenzhen. Information on socio-demographic characteristics, children’s annual screen time since birth, and hyperactive behaviors (measured by the Conners Parental Symptom Questionnaire) was collected through self-administered structured questionnaires completed by the primary caregiver. A series of logistic regression models assessed the association between screen time and hyperactive behaviors.

**Results:**

The average daily screen time of children under the age of 3 years was 55.83 ± 58.54 min, and screen time increased with age. Binomial logistic regression analysis found that the earlier the screen exposure, the greater the risk of hyperactive behaviors. Using binary logistic regression model, after controlling for confounding factors, the study found that more screen time was more associated with hyperactive behaviors. For children aged 0–3 years with daily screen time exceeding 90, 120, 150, and 180 min, the risk values for hyperactive behaviors were 1.98 [95% confidence interval (CI): 1.05, 3.78), 2.71 (95%CI:1.38, 5.30), 3.17 (95% CI: 1.50, 6.65), and 4.62 (95% CI: 2.45, 8.71)], respectively.

**Conclusion:**

Early screen exposure may be associated with hyperactive behaviors in children under the age of 3 years. More than 90 min of screen time per day in children under 3 years was associated with hyperactive behaviors. The findings support the importance of screen time interventions for children under 3 years.

## Introduction

Attention-deficit hyperactivity disorder (ADHD) is one of the most common neurodevelopmental disorders that often affects an individual’s educational achievement and peer relationships, and is associated with an increased risk of adverse life events, such as antisocial activity and illicit drug use (1). Hyperactive behaviors are the main clinical manifestations of ADHD (2) and are one of the most common neurobehavioral conditions in 3-year-old children. In addition, hyperactive behaviors also exert long-term economic burden upon families and the entire country (3). Hyperactive behaviors can emerge in early childhood and continue into adulthood, which may lead to a lifetime dysfunction without effective treatment or prevention (4). The causes of hyperactive behaviors are still unknown, but results from a wide range of epidemiological studies have established a relationship with prenatal exposure to environmental factors. Screen choice has increased significantly in the last two decades (5), and there is growing concern that screen time may have a negative impact on mental health (6). The effects of screen exposure on early life and the boundary between screen time and hyperactive behaviors are well worth investigating.

A Canadian study found that children aged 3–5 years who spent more than 2 h per day in front of a screen had a 7.7 times higher risk of developing hyperactive behaviors than those who spent less than 0.5 h a day in front of a screen (7). A cross-sectional study of school-age children has shown that an increased TV time is associated with hyperactive behaviors (8). Screen time should be considered a risk factor for ADHD symptoms, according to a study examining sedentary behavior in adolescents (9). One review looked at the association between screen time and hyperactive behaviors over nearly four decades and found a slightly significant statistical association (10). Some studies have used theoretical models to explain the relationship between screen exposure and hyperactive behaviors, and estimated that the rapid conversion of electronic screen images would increase children’s excitability and cause hyperactive behaviors (11). Previous studies have mostly focused on children and adolescents, and there have been few studies on the relationship between screen time and hyperactive behaviors in children under the age of 3. There were no studies on the threshold between screen time and hyperactive behaviors in children under the age of 3. However, we do know that early screen time has a greater impact on children’s neuropsychological development because the brains of children under 3 years of age will be undergoing rapid development (12, 13). Therefore, we used data from the Chinese Longhua Children Cohort Study (LCCS) to investigate the association of early screen exposure and longer screen time on hyperactive behaviors in children under 3 years of age.

## Materials and methods

### Study population

Participants in this study were from the 2019 to 2020 LCCS survey. The LCCS is an ongoing prospective cohort study of preschoolers in Longhua, Shenzhen, China, which aims to assess the influence of the family environment on early childhood psychobehavioral development. During 2019–2020, children aged around three and their parents were enrolled from 250 kindergartens in Longhua District of Shenzhen, China. Exclusion criteria were children with serious physical illness or mental disorder. A total of 51,520 questionnaires were sent out from 2019 to 2020, and 47,113 were returned with a recovery rate of 91.45%. The sample size of the study was 42,841 questionnaires (excluding 2,111 that provided incomplete exposure and outcome information). This study was approved by the Ethics Committee of the Longhua Maternal and Child Health Hospital of Shenzhen (ethics license number: 2016102501) and the School of Public Health of Sun Yat-sen University (ethics license number: 2015–016). All participants provided informed written consent to participate in this study.

### Data collection

To create a more standardized survey, members of the research team trained kindergarten principals and doctors twice a year. Each kindergarten held a parents’ meeting in school and invited primary caregivers of 3-year-old children (generally the mother) to attend. After obtaining informed consent from the primary caregiver, a questionnaire survey was conducted. This survey used the electronic questionnaire of WeChat small program following other studies (14, 15) WeChat as the product of Tencent company is a widely used social communication app with more than 1.2 billion users in China and WeChat small program is easily obtained and spread in WeChat, which has an excellent user experience. The electronic questionnaire was set up in logical conditions, and if missed, the system would send automatic prompts. Information was obtained via questionnaire which contained screen time for children under 3 years of age, hyperactive behavior status, general information about the children (such as sex, age), parents’ socio-demographic characteristics (including age at birth, educational level, monthly family income, and marital status).

### Measurement of exposure to electronic screen (primary exposure variable)

Caregivers of children were investigated retrospectively by questionnaire. The aim was to investigate screen exposure and screen time in children under 3 years of age. The following is an excerpt from the questionnaire for 0–1-year-olds (see Table 1).

**TABLE 1:** Questions and options for screen exposure (0–1 years of age as an example).

No.	Questions	Options
Q1	Did your child watch television at age 0–1 years?	A = “no” B = “yes”
Q1.1	If “yes” was chosen for (1.1), how long on average did your child spend watching television per day at age 0–1 years?	Minutes
Q2	Did your child use handheld electronic devices (e.g., mobile phone, tablet computer PAD, game console, etc.) at age 0–1 years?	A = “no” B = “yes”
Q2.1	If “yes” was chosen for (2.1), how long on average did your child spend using handheld electronic devices (e.g., mobile phone, tablet computer PAD, game console, etc.) per day at age 0 1 years?	Minutes
Q3	Did your child watch computer, notebook at age 0–1 years?	A = “no” B = “yes”
Q3.1	If “yes” was chosen for (3.1), how long on average did your child spend watching computer, notebook per day at age 0–1 years?	Minutes

For example, if YES is selected for Q1, Q1.1 problems will be displayed, otherwise Q1.1 will be hidden.

### Measurement of hyperactive behaviors (outcome variable)

Children’s hyperactive behaviors were measured using the hyperactivity index (HI) of Conners’ Parent Rating Scale-48 item version (CPRS-48). CPRS-48 is an internationally transmitted and validated screening tool for assessing behavioral difficulties in children aged 3–16 years (16). This tool has been translated into Chinese and has shown good reliability and validity (17). CPRS-48 Cronbach α coefficient was 0.83, the composite reliability was 0.94, and the average variance extracted was 0.77. The results of a confirmatory factor analysis revealed that HI had good construct validity (most of the items had factor loadings above 0.60, CFI = 0.91, GFI = 0.95, RMSEA = 0.08) (18). These findings indicated that HI is a reliable and validated tool for the measurement of Chinese children’s hyperactive behaviors. HI is comprised of 10 items. The specific contents of the 10 items are as follows: excitable and impulsive; cries easily or often; restless in a squirmy sense; restless, always up, and on the go; destructive; fails to finish things; high distractibility or low attention span; quick and drastic mood changes; easily frustrated; and disturbs other children. Each item is rated on a scale of 0–3 depending on the extent to which each statement is true of the children’s behaviors, i.e., never (for a score of 0), sometimes (score of 1), often (score of 2), and frequently (score of 3). The measurement of hyperactive behaviors was originally a continuous variable ranging between 0 and 3, where a higher score indicated a higher level of hyperactive behavior. The items were summed and then divided by 10 to get the mean score. The HI score ≥ 1.5 was used as the cutoff for establishing hyperactive behaviors in Chinese children (19). It was also treated categorically in previous literature, using a cut-off score of 1.5 to identify the children with and without hyperactivity behaviors (20). In the current study, the measurement of hyperactive behaviors was treated in both categorical and continuous formats for analysis.

### Covariates

The following confounding covariates were included in the analysis: child’s age and sex, parents’ age at child’s birth, parents’ education level, family’s monthly income, and parental marital status.

### Statistical analysis

For continuous variables, we reported the mean and standard deviation (SD), and for categorical variables, numbers and proportions were presented. Chi-square tests and analysis of variance were applied to compare the socio-demographic characteristics between children with and without hyperactive behaviors. To examine the associations between exposure to electronic screens (i.e., initial age of screen exposure, daily average screen time) and hyperactive behaviors, a series of logistic regression models were fitted after adjusting for the covariates. Moreover, we conducted further analyses to probe into the sensitive period and cumulative effect between exposure to electronic screen and hyperactive behaviors. Firstly, the age sensitivity of screen time was analyzed. Binomial logistic regression analyses were used to model the association between early screen exposure and hyperactive behaviors based on annual exposure (Yes) and non-exposure (No) across the children’s three age groups of 0–1, 1–2, and 2–3 years. Secondly, referring to recommendations for children’s screen time in the United States, Canada and previous studies of screen time on children’s behavioral boundaries of the recommendations for children’s screen time (21, 22), we divided the average daily screen time of children into eight subgroups (no screen exposure, < 30 min, 31–60 min, 61–90 min, 91–120 min, 121–150 min, 151–180 min, > 181 min). By finely dividing screen time, we can more accurately guide parents in the proper use of electronic screens. Binary logistic regression was used to analyze the relationship between screen time and hyperactive behaviors in each subgroup. The results were presented as odds ratio (OR) with 95% confidence intervals (*CI*). Statistical significance was set at a two-tailed test with *P* < 0.05. Data management and statistical analysis were performed using Statistical Package for the Social Sciences (version 25.0; SPSS Inc., Chicago, IL, USA).

## Results

### Social characteristics and hyperactive behaviors

Table 2 shows an overview of the sociodemographic characteristics of the participants. Of the 42,841 children in the study, 345 (0.81%) had hyperactive behaviors. The number of male participants 0.93% was higher than that of female participants 0.68% (*P* < 0.01). This result supports conclusions of previous studies (23, 24). Low educational level of parents, low economic income, and single-parent families are risk factors for hyperactive behaviors. See Table 2 for details.

**TABLE 2:** Social characteristics and children’s hyperactive behaviors.

Characteristics	Total (*N* = 42,841)	Hyperactive behaviors		χ^2^/t	*P-value*
		No (*N* = 42,496)	Yes (*N* = 345)		
Child’s age [mean ± SD (years)]	42,841	3.28 ± 0.61	3.25 ± 0.58	1.08	0.27
**Child’s sex [*n* (%)]**					
Male	22,901	22,690 (99.07)	211 (0.93)	8.29	<0.01
Female	19,940	19,806 (99.32)	134 (0.68)		
**Single child status [*n* (%)]**					
Yes	26,014	25,831 (99.29)	183 (0.71)	8.60	<0.01
No	16,827	16,665 (99.03)	162 (0.96)		
Maternal age at child s birth [mean ± SD (years)]	42,481	28.11 ± 3.10	27.22 ± 3.44	28.02	<0.01
Paternal age at child s birth [mean ± SD (years)]	42,481	30.83 ± 4.90	29.47 ± 4.83	26.38	<0.01
**Maternal education level [*n* (%)]**					
Junior high school or lower	5,844	5,778 (98.87)	66 (1.13)	12.78	<0.01
High school	9,360	9,275 (99.09)	85 (0.91)		
College	26,163	25,978 (99.29)	185 (0.71)		
Master’s degree or above	1,474	1,465 (99.39)	9 (0.61)		
**Paternal education level [*n* (%)]**					
Junior high school or lower	4,930	4,867 (98.72)	63 (1.28)		
High school	8,875	8,799 (99.14)	76 (0.86)		
College	26,682	26,491 (99.28)	191 (0.72)		
Master’s degree or above	2,354	2,339 (99.36)	15 (0.64)		
**Monthly household income [*n* (%))]**					
≤¥10,000	9,592	9,481 (98.84)	111 (1.16)	21.13	<0.01
¥10,000–20,000	15,194	15,078 (99.23)	116 (0.77)		
¥20,000–30,000	9,163	9,098 (99.29)	65 (0.71)		
>¥30,000	8,892	8,839 (99.40)	53 (0.60)		
**Parental marital status [*n* (%)]**					
Married	41,556	41,231 (99.21)	325 (0.78)	12.20	<0.01
Unmarried/divorced/	1,228	1,210 (98.53)	18 (1.47)		
Widowed/remarried	57	55 (96.49)	2 C3.51)		

χ^2^; was chi-square test: t, Student’s *t*-test; SD, standard deviation; N (%), quantity (proportion).

### Distribution of screen time by age for children under 3 years of age

**FIGURE 1:**
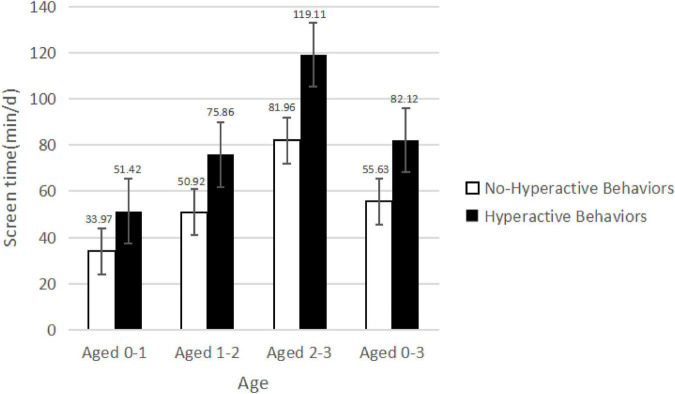
Comparison of screen time at different ages between children with hyperactive behaviors and children without hyperactive behaviors.

Figure 1 shows the comparison of screen time of children with hyperactive behaviors and children without hyperactive behaviors at different ages. Children with hyperactive behaviors have higher screen time than children without hyperactive behaviors at every age. Screen time for children under 3 years of age increased with age. Screen time for children under the age of 1 year was 34.12 ± 53.87 min. Screen time for children under 2 years old was 51.12 ± 65.77 min. Screen time for children under 3 years of age was 82.27 ± 83.47 min. Screen time for children under 3 years of age was 55.83 ± 58.54 min.

### Effects of early screen exposure on children under 3 years of age

Table 3 shows that screen exposure at ages 0–1 and 1–2 is associated with hyperactive behavior. Screen exposure at 2–3 years of age was not significantly associated with hyperactive behavior (Model 1). The binomial logistic regression model (Model 2) included screen exposure and hyperactivity in three different age groups and found that 0–1 year old screen exposure was more associated with hyperactivity. The results held after adjusting for relevant covariates (model 3).

**TABLE 3:** The relationship between early screen exposure and hyperactive behaviors (*N* = 42,841).

Annual exposure (yes) or non-exposure (no)	Model 1^‡^ OR (95% *CI*)	Model 2^§^ OR (95% *CI*)	Model 3^¶^ OR (95% *CI*)
Age 0–1	1.66 (1.32–2.08)***	1.51 (1.18–1.95)**	1.48 (1.15–1.91)**
Age 1–2	1.55 (1.20–2.01)**	1.24 (0.92–1.69)	1.23 (0.90–1.66)
Age 2–3	1.31 (0.92–1.87)	1.08 (0.73–1.58)	1.05 (0.71–1.55)

^‡^The first model independently analyzed the association between screen exposure and hyperactive behaviors at ages 0–1, 1–2, and 2–3 years.

^§^The second model included screen exposure at each age into a binomial logistic regression model to analyze the association between screen exposure at each age and hyperactive behaviors.

^¶^The third model analyzed the association between screen exposure and hyperactivity at each age after adjusting for child’s sex, number of children in the family, maternal and paternal age at child’s birth, maternal and paternal education level, monthly household income, and parental marital status.

*CI*: Confidence intervals. **P < 0.01; ***P < 0.001.

### Chi-square test and conditional logistic regression analysis of screen time and hyperactive behaviors (primary outcome)

As shown in Table 4, there was a statistical difference between screen time and the chi-square test of hyperactive behaviors. After controlling for confounding factors using an unconditional logistic regression analysis, the risk of hyperactive behaviors increased as average daily screen time increased. After average daily screen time exceeded 90 min, the risk of hyperactivity increased rapidly with increased screen time.

**TABLE 4:** Chi-square test and conditional logistic regression analysis of screen time and hyperactive behaviors.

Daily screen time by age 0–3	No. of children	Cases (N%)	AOR (95% *CI*)	χ^2^/*P-value*
No screen exposure	2,952	13 (0.44)		84.28/ < 0.001
Screen time < 30 min	12,284	84 (0.68)	1.59 (0.88–2.87)	
Screen time 31–60 min	12,071	76 (0.63)	1.41 (0.78–2.55)	
Screen time 61–90 min	7,329	54 (0.74)	1.57 (0.78–2.55)	
Screen time 91–120 min	3,613	35 (0.97)	1.98 (1.05–3.78)*	
Screen time 121–150 min	1,919	26 (1.35)	2.71 (1.38–5.30)**	
Screen time 151–180 min	974	16 (1.64)	3.17 (1.50–6.65)**	
Screen time > 181 min	1,699	41 (2.41)	4.62 (2.45–8.71)***	

AOR, Adjusted odds ratio. OR with adjustment for child’s sex, number of children in the family, maternal and paternal age at child’s birth, maternal and paternal education level, monthly household income, and parental marital status. CI, Confidence intervals; Ref, Reference. *P < 0.05; **P < 0.01; ***P < 0.001.

## Discussion

### Screen time for children under the age of 3 years

With the rapid development of technology and economy, almost every family has electronic screen products. Many caregivers regard electronic products as “electronic babysitters” to reduce their children’s disturbance (25). In addition, the constant emergence of educational video programs has increased screen exposure for children under 3 years of age (26). Our research has found that screen time increases as children age. Low parental education is an important risk factor for screen exposure, which is consistent with previous studies (27, 28). This suggests that the most important intervention group to reduce screen time in children is the population with low educational level. The survey found that 1-year-olds in the region spent 34 min, 2-year-olds spent 51 min and 3-year-olds spent 82 min in front of a screen.

The proportion of children with screen exposure before 1 year old was as high as 57.1%, while the proportion of children without screen exposure before 2 years old was only 22.40%. This significantly exceeds the screen guidelines recommended by the American Academy of Pediatrics in 2016. The screen guidelines recommend that children under 18 months should not use screens except for chatting, that children between 18 and 24 months should have limited exposure to screens, and that children between 2 and 5 years old should not spend more than 1 h a day on screen (29).

### Effects of screen time on hyperactive behaviors

Our analysis of big data from birth cohorts found that the longer the children’s daily screen time, the greater the risk of an increase in hyperactive behaviors. Children with hyperactive behaviors had more screen time. We found that the earlier the age of screen exposure, the more associated with hyperactive behaviors. Children under the age of 3 who spend more than 90 min a day in front of screens was associated with hyperactive behaviors, which is largely consistent with recommendations from the American Academy of Pediatrics’ 2016 screen guidelines (29).

A Canadian study found that children aged 3–5 who watched electronic screens for more than 2 h a day were more likely to develop hyperactive behaviors than those who watched screens for just 30 min a day (7). Yet a British study reports that screen time is not an increased risk factor for behavioral problems in children as young as 5 (30). The difference may be related to the weaker effect of screen time on older children. This study helps clarify this question. Our study found that the earlier the exposure to electronic screens, the more likely it was to associate with hyperactive behaviors. It is possible that in terms of developmental susceptibility, young children are more susceptible to the effects of electronic screens than children and adolescents (31). Young children are less able to control their arousal level when watching electronic screens, and the effects of electronic screens may be stronger in young children than in children and adolescents (32).

Previous studies have focused on the link between screen time and hyperactive behaviors in children older than 3, while few studies have looked at the effect of screen time on hyperactive behaviors in children younger than 3. A Japanese study found that ADHD at 30 months was positively correlated with time spent watching TV at 18 months, while prosocial behavior was negatively correlated with time spent watching TV, even after adjustment. However, at 30 months, there was no significant difference in the strength and difficulty questionnaire subscale based on daily TV viewing time (33). This study is consistent with our own evidence that screen time is developmentally sensitive to the effects of hyperactive behaviors. The study also demonstrated an association between screen time and hyperactive behaviors, but this study had a high rate of lost to follow-up and had a small sample size. We used an empirically validated result to measure hyperactive behaviors in young children. The large sample size allowed us to observe the association between screen time and hyperactive behaviors, controlling for multiple confounding factors.

In 2018, a review of screen time in ADHD in the recent 40 years published in the proceedings of the National Academy of Sciences, related research were analyzed, regarding its the potential mechanism (10, 11): First, it was assumed that it was based on the excitation reaction condition allowing the child to repeatedly update electronic screen orientation response and cause increased wakefulness. Accustomed to being on a fast pace, children’s baseline arousal levels may decrease, eventually leading to hyperactive behaviors. Second is the scanning and diversion hypothesis, which is based on the role of cognitive response states and believes that watching electronic screens prevents children from developing attention skills (34, 35). Because children with hyperactive behaviors have difficulty engaging in developmentally appropriate play tasks that require sustained attention, children may prefer screen devices to play-based activities because they tend to offer more multisensory and diverse stimulation (36). In addition, for children with hyperactive behaviors, parents may give them more screen time, which seems to reduce hyperactive behaviors. However, as screen time increases, these children may be more likely to miss out on real-world learning opportunities and may replace developmentally beneficial interactions, which may further exacerbate hyperactive behaviors (37, 38).

The main advantage of our study is the detailed breakdown of screen time, which gives a threshold for the effect of screen time on hyperactive behaviors. We also analyzed several potential confounding factors through a large sample of data, further supporting the relationship between screen time and hyperactive behaviors in children under 3 years of age. We also looked at earlier screen exposure, with greater risk of hyperactive behaviors. Our study reminds parents to control screen time in early childhood. More than 90 min of screen time per day may be associated with hyperactive behavior. This study has limitations. First, although the sample size of this study is large, longitudinal studies are still needed to further verify the hypothesis of causality. Second, the data collected on electronic screen exposure is retrospective and relies on parental reporting accuracy, which may be prone to recall bias. Third, this study only assessed screen time, and screen content may modulate the effects of screen exposure (39, 40). Fourth, this study was mainly performed in Longhua District of Shenzhen. Parents may have regional characteristics in terms of education level, which cannot fully represent the situation in China. Further nationwide research is needed.

## Conclusion

Overall, our study found that early screen exposure may be associated with hyperactive behaviors in children under 3 years of age. More than 90 min of screen time per day in children under 3 years was associated with hyperactive behaviors in 3-year-olds. The findings support the importance of screen time interventions for children under 3. This study provides preliminary guidance for screen time use in children under 3 years of age.

## Data availability statement

The original contributions presented in this study are included in the article/supplementary material, further inquiries can be directed to the corresponding author/s.

## Ethics statement

This study was approved by the Ethics Committee of the Longhua Maternal and Child Health Hospital of Shenzhen (ethics license number: 2016102501) and the School of Public Health of Sun Yat-sen University (ethics license number: 2015–016). Written informed consent to participate in this study was provided by the participants or their legal guardian/next of kin.

## Author contributions

DL and JJ: conceptualization, project administration, supervision, and validation. J-BW and J-YZ: data curation. J-BW and DL: formal analysis. J-BW and W-KY: funding acquisition. X-BH and Y-FZ: investigation. S-YQ and XW: methodology. J-BW: resources. G-MW: software. J-BW and X-NY: visualization and writing – original draft and review and editing. All authors contributed to the article and approved the submitted version.
